# TFIIB Co-Localizes and Interacts with α-Tubulin during Oocyte Meiosis in the Mouse and Depletion of TFIIB Causes Arrest of Subsequent Embryo Development

**DOI:** 10.1371/journal.pone.0080039

**Published:** 2013-11-14

**Authors:** Hui Liu, Feng-Xia Yin, Chun-Ling Bai, Qi-Yuan Shen, Zhu-Ying Wei, Xin-Xin Li, Hao Liang, Shorgan Bou, Guang-Peng Li

**Affiliations:** The Key Laboratory for Mammalian Reproductive Biology and Biotechnology, Ministry of Education, Inner mongolia University, Hohhot, China; Institute of Zoology, Chinese Academy of Sciences, China

## Abstract

TFIIB (transcription factor IIB) is a transcription factor that provides a bridge between promoter-bound TFIID and RNA polymerase II, and it is a target of various transcriptional activator proteins that stimulate the pre-initiation complex assembly. The localization and/or attachment matrix of TFIIB in the cytoplast is not well understood. This study focuses on the function of TFIIB and its interrelationship with α-tubulins in a mouse model. During oocyte maturation TFIIB distributes throughout the entire nucleus of the germinal vesicle (GV). After progression to GV breakdown (GVBD), TFIIB and α-tubulin co-localize and accumulate in the vicinity of the condensed chromosomes. During the MII stage, the TFIIB signals are more concentrated at the equatorial plate and the kinetochores. Colcemid treatment of oocytes disrupts the microtubule (MT) system, although the TFIIB signals are still present with the altered MT state. Injection of oocytes with TFIIB antibodies and siRNAs causes abnormal spindle formation and irregular chromosome alignment. These findings suggest that TFIIB dissociates from the condensed chromatids and then tightly binds to microtubules from GVBD to the MII phase. The assembly and disassembly of TFIIB may very well be associated with and driven by microtubules. TFIIB maintains its contact with the α-tubulins and its co-localization forms a unique distribution pattern. Depletion of *Tf2b* in oocytes results in a significant decrease in TFIIB expression, although polar body extrusion does not appear to be affected. Knockdown of *Tf2b* dramatically affects subsequent embryo development with more than 85% of the embryos arrested at the 2-cell stage. These arrested embryos still maintain apparently normal morphology for at least 96h without any obvious degeneration. Analysis of the effects of TFIIB in somatic cells by co-transfection of BiFC plasmids pHA-*Tf2b* and pFlag-*Tuba1α* further confirms a direct interaction between TFIIB and α-tubulins.

## Introduction

Oocyte nuclear-associated factors are critical for fertilization and somatic cell nuclear reprogramming. The oocyte acquires its reprogramming capacity in the early fetal follicle. The reprogramming capacity does not reach its highest potential until the late growth phase when a fully-grown germinal vesicle (GV) is formed [1-3]. There are two phases of transcriptional activation during oocyte meiotic maturation in the mouse. The first phase takes place from the time of oogenesis when a large number of factors are required and accumulated for meiotic maturation and early embryonic development, to the time when chromosomal condensation is completed at the late GV stage [4,5]. Critical transcription factors and other regulators separate from chromatin in the nucleus over a long period of time, and then re-associate with chromatin shortly after the pronucleus is formed [6,7]. The second phase takes place when the pronucleus is formed after fertilization. Transcription factors (TFs) then enter the nucleus and rebind to the chromatin to initiate the transcriptional process. During the initiation of transcription, the transcription factor IID (TFIID) binds to a TATA box core promoter, which is then stabilized by the transcription factor IIB (TFIIB) [7-9]. The initiation of zygotic transcription during maternal zygotic transition (MZT) begins with the assembly of the pre-initiation complex on the promoter [10,11]. Transcriptional activity is competitively regulated by the chromatin and the assembly of the transcriptional machinery [12]. TFs in the mouse are disrupted by physical connections of chromatin and transcription factors, and the maternal transcription program is removed to a functional level [13]. The interference of TF expression prevents oocyte maturation and interferes with embryogenesis [2,14,15].

Nuclear and cytoplasmic proteins are involved in the meiotic processes from oocyte maturation to early embryonic development. Microtubules (MTs) and microfilaments (MFs) that form the cytoskeleton are directly involved in the formation of meiotic spindles. Spindles are dynamic cellular structures and their formation and morphological changes are achieved by MTs and MFs, and by various motor proteins associated with chromosomes and MTs [16-18]. Polymerization of MTs and MFs play key roles in the regulation of chromosome alignment and segregation, the movement of nuclear material from a central position to the cortical area and the emission of the first polar body (Pb1) [16,18]. 

 This study examines the physical relationships between microtubules and TFIIB using immunocytochemical staining techniques, interruption of the microtubule assembly, the knockdown and depletion of *Tf2b* with RNA interference and antibody injection. We examined the effects of TFIIB disruption on oocyte nuclear and cytoplasmic maturation and subsequent embryo development. The microtubule-driven dynamic assembly and disassembly of TFIIB from chromatin/chromosome is a major emphasis of this study. 

## Materials and Methods

### Ethics statement

All procedures used in this study are approved by the Inner Mongolia University Animal Care and Use Committee.

### Chemicals

Chemicals were purchased from Sigma Chemical Co. (St. Louis, MO) unless otherwise indicated. Primers were synthesized by Takara Biotechnology Dalian Co. Ltd (Dalian, China), and sequencing assays were performed by Invitrogen Life Technologies Corporation. Antibodies were purchased from Santa Cruz Biotechnology Inc (Santa Cruz, California). 

### Collection and maturation of oocytes *in vitro*

BDF1 mice were provided by the SPF (specific pathogen free) Animal Breeding Center of Inner Mongolia University. Immature oocytes were collected from ovaries of 5-6 week-old female mice in M2 medium (Millipore, USA) supplemented with 60 µg/ml penicillin and 50 µg/ml streptomycin at 48h of PMSG injection. Only cumulus-oocyte complexs (COCs) with an obvious germinal vesicle (GV) were selected and cultured in modified Krebs-Ringer's bicarbonate solution (TYH) (Kito et al., 2004) medium supplemented with 4 mg/ml BSA,1 IU/ml PMSG, 1% non-essential amino acids and 5% FBS (Gibco) under mineral oil at 37°Cin a humidified 5% CO_2_ incubator. COCs used for siRNA (small interfering RNA) injection were mechanically denuded at the GV stage and then cultured in TYH medium for 1-1.5h in the presence of 0.2 mM 3-isobutyl-1-methylxanthine (IBMX), a phosphodiesterase inhibitor used to suppress the occurrence of germinal vesicle breakdown (GVBD). 

### Oocyte parthenogenetic activation and *in vitro* development

MII oocytes were obtained by superovulating mice with PMSG, followed 48 h later with hCG. Fourteen hours after treatment, the mice were sacrificed and COCs were collected. Cumulus cells were removed from the oocytes by exposure to 300 μg/ml hyaluronidase in M2 medium. The denuded oocytes were rinsed gently in Ca^2+^-free KSOM medium. Oocytes injected with siRNA and the non-treated control were activated with 10 mM SrCl_2_ and 5 μg/ml cytochalasin B in Ca^2+^-free KSOM for 5 h at 37°C in 5% CO_2_ in air. The activated oocytes were incubated in 50 μl drops of KSOM covered with a thin layer of oil at 37°C in 5% CO_2_ containing air for 4 days.

### Immunofluorescence staining, chromosome spreads and image analysis

Oocytes were rinsed three times in PBS with 0.3% BSA, fixed with 1% paraformaldehyde and permeabilized with 0.2% Triton X-100 in PBS simultaneously overnight at 4°C, followed by washing thoroughly in PBS containing 0.3% BSA. After being blocked in PBS plus 3% BSA for 1-2 h at room temperature, the oocytes were incubated at 4°C overnight with primary TFIIB antibodies. TFIIB was applied at a dilution of 1:400 while anti-mouse-α-tubulin (Sigma) was diluted 1:1000. After five washes in PBS containing 0.3% BSA, oocytes were then labeled with a secondary antibody diluted 1:500 for 2 h at room temperature. For double staining of TFIIB and α-tubulin, after TFIIB staining (the secondary antibody was 1:400 FITC -conjugated antibody), the oocytes were blocked again in PBS plus 3% BSA for 1 h at room temperature. The oocytes were then stained with 1:400 goat-anti-rabbit-R-IgG, after which they were washed thoroughly and stained with 10 μg/ml Hoechst 33342 for 5 min. After extensive washing, oocytes were mounted on glass slides and examined with an Olympus Fluoview 1000 Confocal Laser-Scanning Microscope. Oocytes and blastocysts were treated with 1% trisodium citrate at room temperature for 10-15 minutes and then fixed quickly with fresh methanol: glacial acetic acid (3:1) on glass slides for 24h to obtain chromosome spreads. Chromosomes were stained with 1% Giemsa for 10 min. Each experiment was repeated three or more times, and a minimum of 20 oocytes were examined in each sample. At least 80% of the samples analyzed displayed consistent results. Instrument settings were kept constant for each replicate. Images were analyzed with an OLYMPUS FLUOVIEW Ver. 1.4a Viewer. 

### Immunoblotting analysis

Immunoblotting was based on procedures previously reported [19]. Briefly, oocytes were treated in sodium dodecyl sulfate (SDS) buffer and heated at 100°C for 4 min, then cooled rapidly for 5 min. The proteins were separated by SDS polyacrylamide gel electrophoresis and electrically transferred to polyvinylidene fluoride membranes. Following transfer, the membranes were blocked in TBST (TBS containing 0.1% Tween 20) containing 5% non-fat milk at 4°C overnight, followed by incubation at 4°C for 12h with either 1:2,000 anti-mouse-α-tubulin antibody or 1:800 rabbit polyclonal anti-TFIIB antibody. After washing 3 times in TBST, 10 min for each washing, the membranes were incubated for 2h at 37°C with 1:1,000 horseradish peroxidase-conjugated goat anti-mouse IgG or horseradish peroxidase-conjugated goat anti-rabbit IgG. The membranes were then processed using an enhanced chemiluminescence detection system.

### Cell culture and transfection

Mouse (BDF1) fibroblasts were obtained from the skin of a mouse fetus at day 13.5, and fibroblasts from passage 3 to passage 4 were used to perform transfections. Embryonic fibroblasts were cultured in DMEM (Dulbecco`s Modified Eagle Medium) supplemented with 10% FBS and 0.2 mM L-glutamine. Lipofectamine 2000 (Invitrogen) was used for plasmids transfection following the guidelines of the manufacturer. Fluorescent signal was examined 48h after transfections. Images were digitally captured using the Nikon Elements Program with a Nikon microscope (KHU, TYO, Japan).

### RNA interference

**Figure 1:**
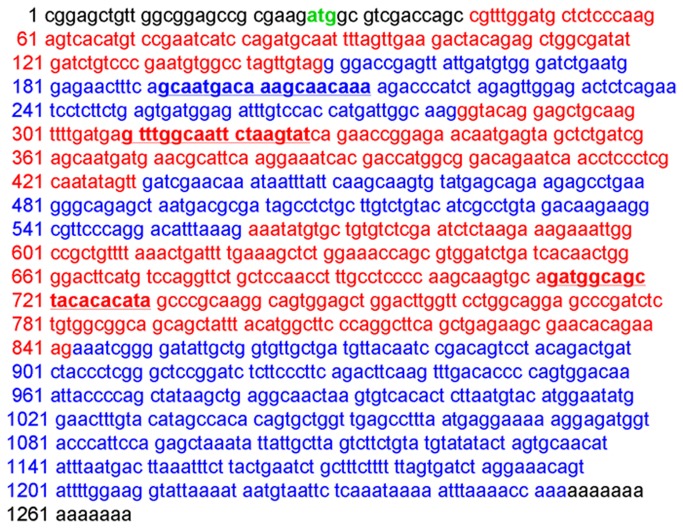
The *Tf2b* mRNA sequence. Green, start codon; red and blue, different exons; underlined and bold, siRNA.

The interference sequences of *Tf2b* were designed via software made available on the Applied Biosystemsg website (http://www.ambion.com/techlib/siRNA_finder.html). As shown in Table 1, three pairs of *Tf2b* siRNA sequences and one negative control siRNA sequence were synthesized by GeneChem (Shanghai, China). The positive control is a *Gapdh* siRNA sequence (purchased from GeneChem). The positions of three pairs of *Tf2b* siRNA sequences in *Tf2b* mRNA sequence are shown in Figure 1.

**Table 1:** Specifications of double-stranded siRNAs of mouse *Tf2b*.

Target gene	Start sites	siRNA sequence
	192	Sense5’-3’: GCA,AUG,ACA,AAG,CAA,CAA,Att
		Antisense5’-3’: UUU,GUU,GCU,UUG,UCA,UUG,Ctt
*Tf2b*	712	Sense5’-3’: GAU,GGC,AGC,UAC,ACA,CAU,Att
		Antisense5’-3’: UAU,GUG,UGU,AGC,UGC,CAU,Ctt
	310	Sense5’-3’: GUU,UGG,CAA,UUC,UAA,GUA,Utt
		Antisense5’-3’: AUA,CUU,AGA,AUU,GCC,AAA,Ctt
Negative control	No matches	Sense5’-3’: UUC,UCC,GAA,CGU,GUC,ACG,Utt
		Antisense5’-3’: ACG,UGA,CAC,GUU,CGG,AGA,Att

For the RNA interference manipulations, three frozen-dried powders of the small interfering RNAs (siRNAs) were diluted to 20μM by RNase free dH_2_O stock solutions and stored at -80°C. The siRNA used in this study was a mixture of three pairs of *Tf2b* siRNA (m- *Tf2b*) [15]. The siRNAs were mixed and diluted 3 times by RNase free dH_2_O prior to microinjection. 5pl of m- *Tf2b* siRNA were microinjected (Eppendorf microinjector, Germany) into GV intact oocytes kept in M2 medium containing 0.2 mM IBMX. The same amount of siRNAs was injected in the positive and negative controls. Pipettes were replaced after fifty oocytes were injected to avoid the potential of false negative results. 

### RNAi analysis by real-time RT-PCR

RNAi oocytes were incubated in TYH medium supplemented with 4 mg/ml BSA,1 IU/ml PMSG, 1% nonessential amino acids and 5% FBS for 0, 6, 10, and 14h. One hundred oocytes from each group were collected and total RNA was isolated using Trizol (Invitrogen) according to the manufacturer’s protocol. The first strand complementary DNA was synthesized by priming with oligo dT primer utilizing the SYBR PrimeScript RT-PCR kit protocol. No more than 500 ng RNA was added to the reverse transcription reaction in a final volume of 10μl. Real time RT-PCR primers were designed and synthesized by Takara Biotechnology Dalian Co. Ltd. (Dalian, China). The *Gapdh* was used as an internal reference gene for normalization of *Tf2b* relative quantifications. The primers used are as follow: 

*Tf2b*, forward, GAAGGCGTTCCCAGGACATTTA, reverse, CGCTGGTTTCCAGAGCTTTCA.

*Gapdh*, forward, TGTGTCCGTCGTGGATCTGA, reverse, TTGCTGTTGAAGTCGCAGGAG. 

Real-time PCR was performed in an ABI prism 7300 Sequence Detection System. The steps were 95°C for 10s, 40 cycles of 95°C for 5s and 60°C for 31s. Analysis of relative gene expression was measured using the 2 (-Delta Delta C (T)) method [20].

### Experimental design

Experiment 1. This experiment was designed to determine the distribution of TFIIB and α-tubulin during oocyte maturation. COCs were cultured for 0, 2, 6, 10 and 14h to establish reference points that correspond to germinal vesicle (GV), germinal vesicle breakdown (GVBD), metaphase I (MI), anaphase/telophase I (AI/TI), and metaphase II (MII) stages. The oocytes were then denuded and processed using TFIIB and α-tubulin antibodies. Subcellular distributions of TFIIB and tubulin were observed under a fluorescence microscope. 

Experiment 2. The results from Experiment 1 show that the distribution of TFIIB and tubulin was co-localized during oocyte meiosis. This experiment was conducted to verify that colcemid-induced disrupted microtubules still maintained a similar distribution pattern as TFIIB. Oocytes at 0, 2, 5, 7, 9, 10, and 14h of maturation were respectively treated with 2 µg/ml colcemid for 30 min. After treatment, the oocytes were stained and analyzed with TFIIB and α-tubulin antibodies.

Experiment 3. To further examine whether decreased levels of TFIIB affect microtubule patterns, TFIIB antibodies were microinjected. Approximately 5 Pl anti-TFIIB (0.5 mg/ml) antibodies were injected into the cytoplasm of fully matured GV oocytes. The oocytes were kept in M2 medium supplemented with 0.2mM IBMX to prevent GVBD during the injection period. The injected oocytes were washed thoroughly with M2 medium and cultured in TYH medium supplemented with 4mg/ml BSA,1IU/ml PMSG,1% NEAA and 5% FBS (Gibco) under mineral oil at 37°C and cultured in 5% CO_2_ containing air in a 90% humidified incubator. Control oocytes were microinjected with a similar volume of PBS. Oocyte spindle patterns were examined by immunofluorescence at various stages of oocyte development.

Experiment 4.To further confirm the correlation between TFIIB and α-tubulin, BiFC plasmids pHA-*Tf2b* and pFlag-*Tuba1α* were constructed and co-transfected into mouse fetal fibroblast cells. Fluorescent signals were detected to confirm the direct correlation of TFIIB and α-tubulin.

Experiment 5. This experiment examined whether treatment of GV oocytes with *Tf2b* RNAi affected oocyte maturation. Oocytes at the GV stage were microinjected with m- *Tf2b* siRNA as described in Experiment 3. The injected oocytes were incubated in maturation medium for 0,6,10 and 14h, and the mRNA and protein levels were examined by Real-time RT-PCR and western blotting. The number of oocytes with the PB1 extruded was counted after maturation for 14h and the resultant oocytes were karyotyped.

Experiment 6. This experiment examined whether *Tf2b* RNAi exposure of GV oocytes affected embryo development. Oocytes at the GV stage were microinjected with m- *Tf2b* siRNA as described in Experiment 3. The injected oocytes were incubated in maturation medium for 14h, and oocytes with extruded PB1 were collected. The matured oocytes were then parthenogenetically activated and cultured to observe development. The chromosomal composition of embryos was then analyzed.

Experiment 7. To observe whether *Tf2b* RNAi of MII oocytes affects subsequent embryo development, MII oocytes were injected with m- *Tf2b* siRNA. The progression of embryo development was then monitored. The chromosomal composition of derived blastocysts was analyzed.

### Statistical analysis

All data (mean ± SE) were analyzed by ANOVA using SPSS software (SPSS Inc, Chicago, IL) followed by the student-Newman-Keuls test. Differences at P<0.05 were considered statistically significant.

## Results

### Co-distribution of TFIIB and α-tubulin during oocyte meiotic maturation

**Figure 2:**
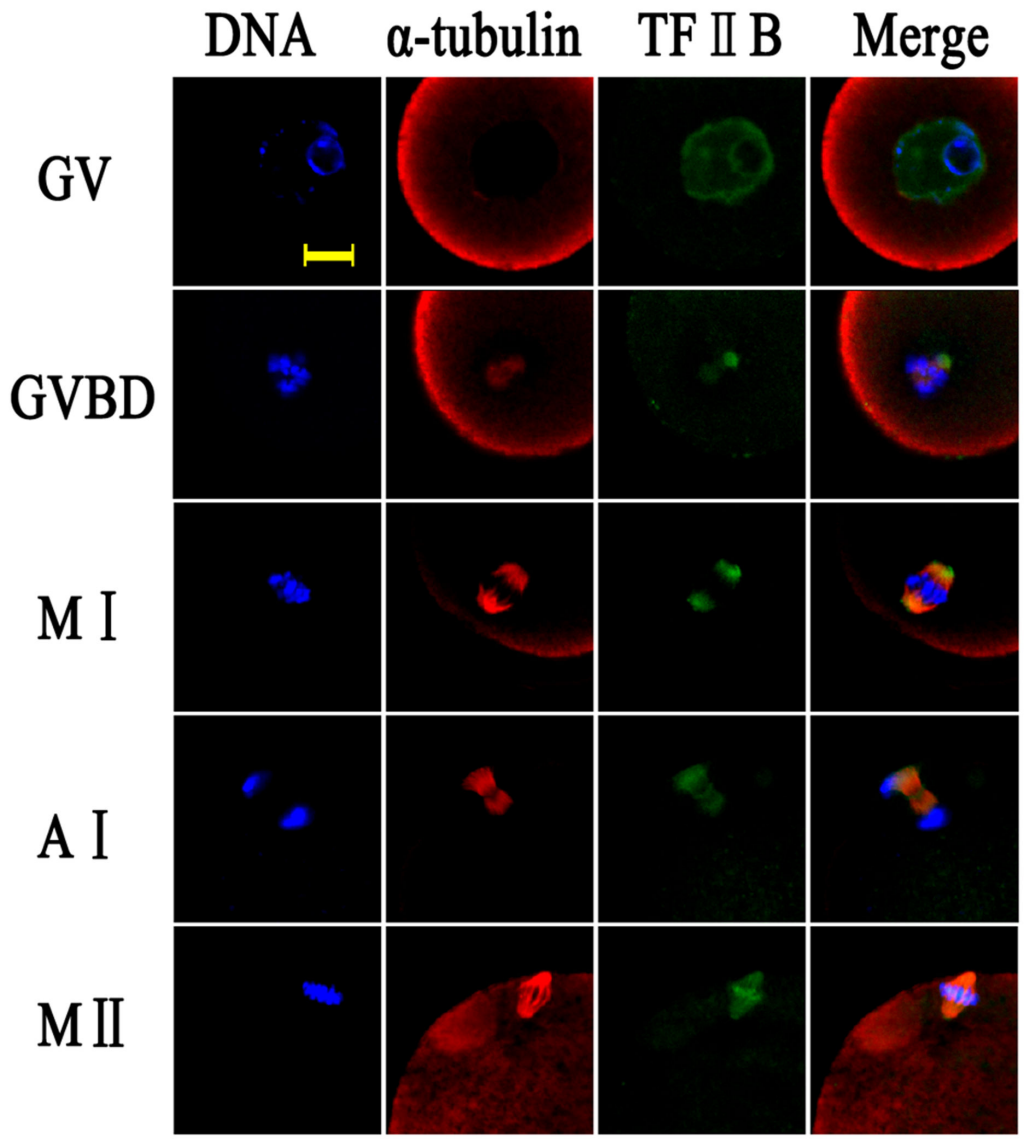
Localization of TFIIB and α-tubulin in maturing oocytes from the germinal vesicle to metaphase II stages. Green, TFIIB; red, α-Tubulin; blue, chromatin; GV, germinal vesicle; GVBD, germinal vesicle breakdown; MI, metaphase I; AI/TI, anaphase I or telophase I; MII, metaphase II. Each sample was counterstained with Hoechst 33342 to visualize the DNA. Bar=10μm.

Oocytes at the GV stage were cultured and processed for immunofluorescent staining at 0, 2, 6, 10 and 14h, respectively. As shown in Figure 2, at the GV stage, TFIIB displayed a general distribution pattern throughout the entire nucleus of the germinal vesicle and was associated with the chromatin [13], while almost no α-tubulin was observed a this stage of development. After progression to GVBD, TFIIB and α-tubulin were co-localized and accumulated in the vicinity of the condensed chromosomes. At the MI stage, when the chromosomes were aligned at the equatorial plate, TFIIB mainly concentrated at the spindle poles; whereas, the α-tubulins formed the spindle and were connected to the chromosomes. At AI/TI, TFIIB was co-localized with α-tubulin and was enriched at the equatorial plate and at the spindle poles. During the MII stage, the TFIIB signals became more concentrated at the equatorial plate, and at the kinetochores.

### Disruption of spindle microtubules by colcemid did not affect the co-localization distribution pattern of TFIIB and α-tubulin

**Figure 3:**
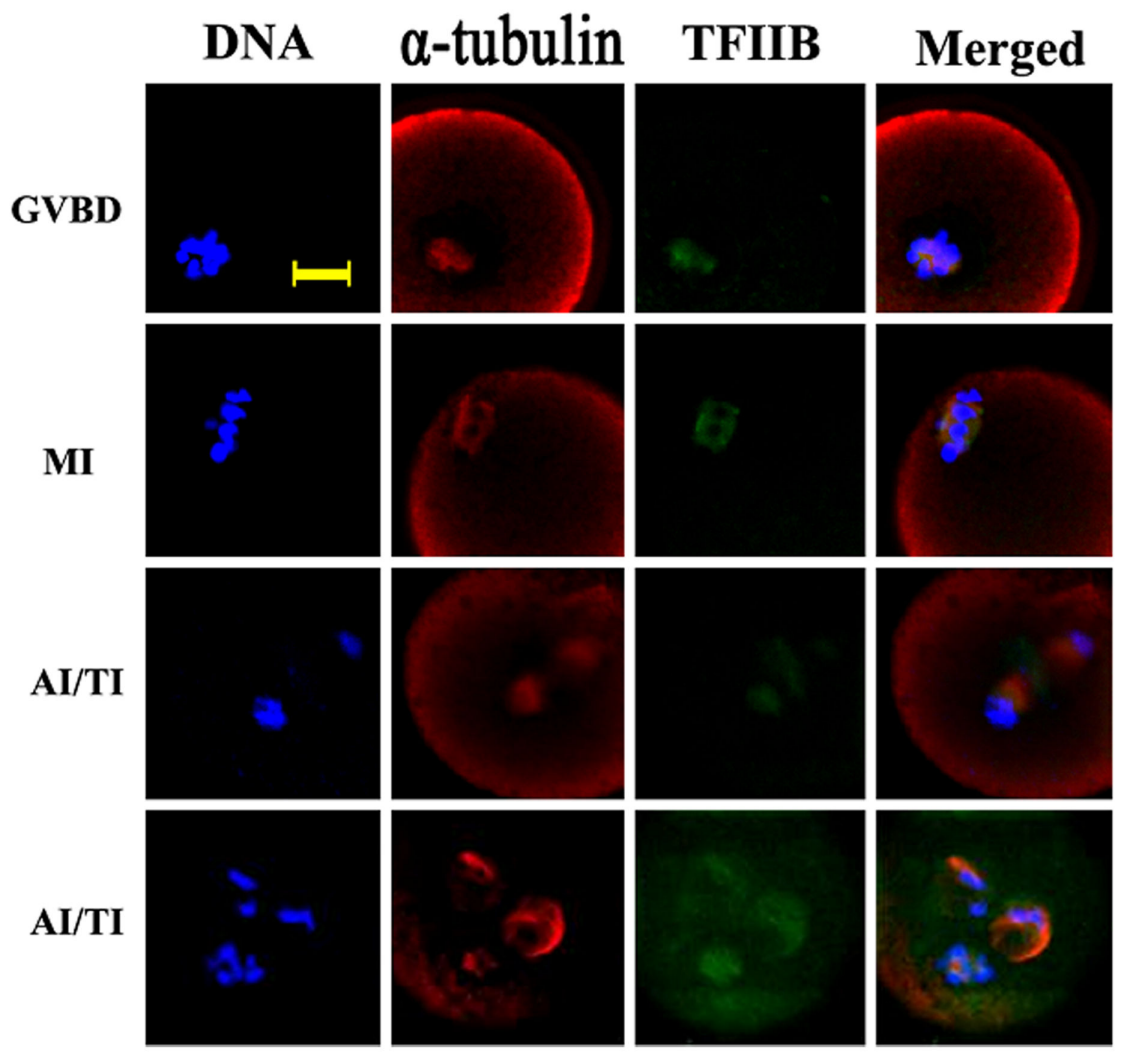
Co-localization of TFIIB and α-tubulin after treatment of oocytes with colcemid. Oocytes at various stages were incubated in 2μg/ml colcemid-containing medium for 30 min and then double stained with antibodies against TFIIB and α-tubulin. Green, TFIIB; red, a-Tubulin; blue, chromatin; GV, germinal vesicle; GVBD, germinal vesicle breakdown; MI, metaphase I; AI/TI, anaphase I or telophase I; MII, metaphase II. Each sample was counterstained with Hoechst 33342 to visualize the DNA. Bar=10μm.

To further clarify the correlation between TFIIB and microtubules, colcemid, an inhibitor of microtubule assembly, was used to treat oocytes during meiosis. Microtubule assembly and spindle formation were disrupted and irregular masses of microtubules were scattered around the chromatin/chromosomes after treatment (Figure 3). The co-localization pattern of α-tubulin and TFIIB from MI to MII was not interrupted (Table 2). 

**Table 2:** Co-localization of TFIIB and α-tubulin after colcemid treatment.

Maturation status		Control			Colcemid (2μg/ml, 30m)		
		No. of oocytes	TFIIB (+)	%	No. of oocytes	TFIIB (+)	%
GV	0h	20	0	0	20	0	0
GVBD	2h	70	47	67.1	75	49	65.3
	5h	53	53	100.0	54	49	90.7
MI	7h	55	55	100.0	55	52	94.5
	9h	52	50	96.2	50	47	94.0
AI-TI	10h	48	48	100.0	48	48	100.0
MII	14h	49	49	100.0	49	48	97.9

### Interference of *Tf2b* resulted in abnormal spindles and irregular chromosomal alignment

**Figure 4:**
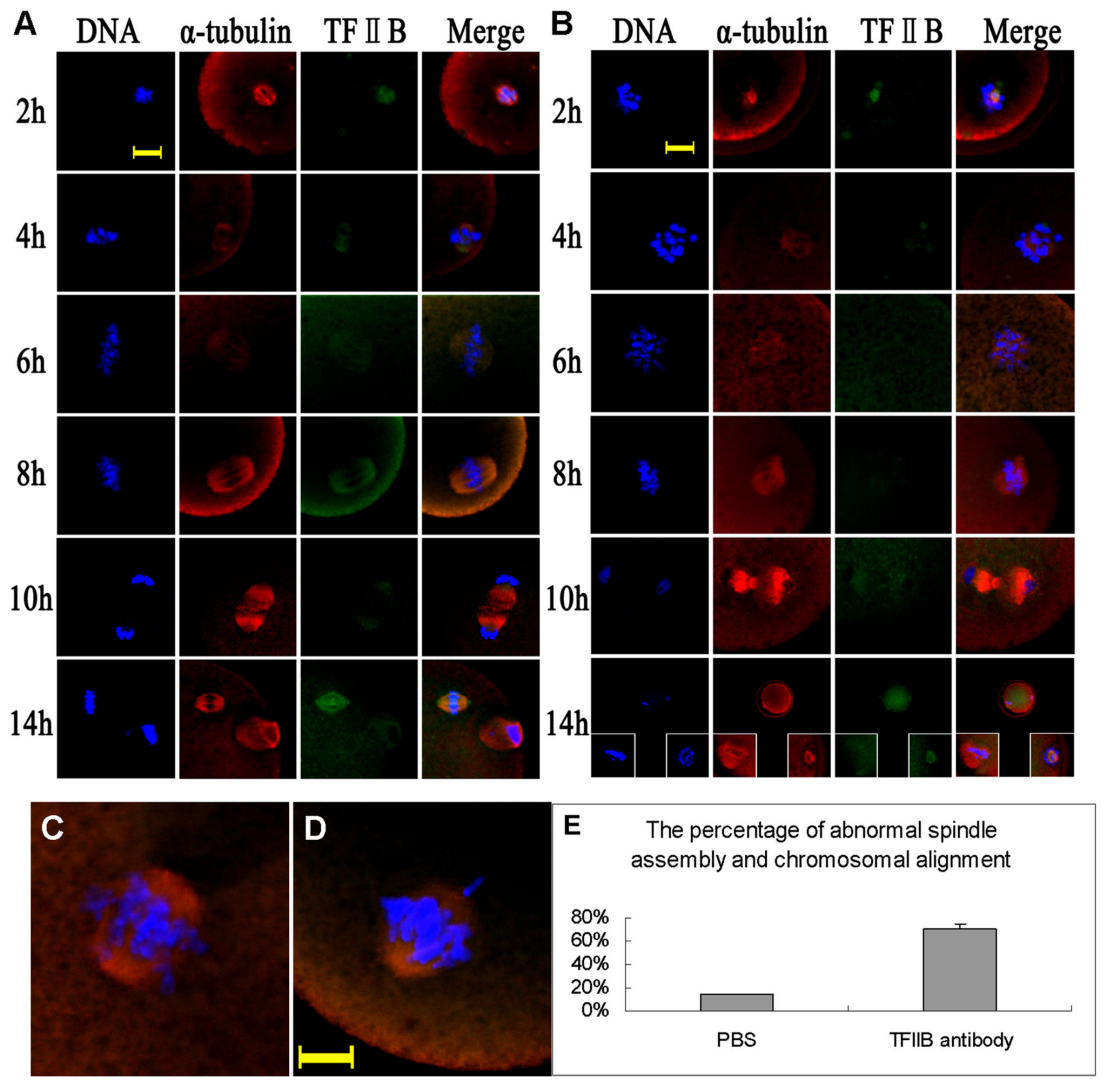
Disruption of TFIIB function induced irregular spindle formation and chromosomal alignment. **A**) Control normal spindles after PBS injection, **B**) irregular spindles induced by TFIIB antibody injection, and **C**, **D**) chromosomal misalignment induced by TFIIB antibody injection, **E**) The percentage of oocytes with irregular spindle formations and chromosomal alignments after TFIIB antibody injection. Bar=10μm.

When TFIIB antibodies were injected into the cytoplasm of GV oocytes, 76% of the oocytes displayed abnormal spindles and the chromosomes were irregularly aligned (Figure 4B, C and D; Figure 4E). In the PBS-injection group, the percentage of oocytes with spindle perturbations and irregular chromosomal alignment was 15% (P<0.05 compared to the treated ones) (Figure 4B and E). The TFIIB signals were strongly positive in PBS-treated or control oocytes (Figure 4A), but almost negligible in the *Tf2b*-depleted oocytes (Figure 4B). The depletion of *Tf2b* resulted in irregular configuration of spindles with altered chromosome alignment.

### BiFC analysis of mitotic cells further revealed the direct interaction between TFIIB and α-tubulin

To further confirm the correlation between TFIIB and α-tubulin, BiFC plasmids pHA-*Tf2b* and pFlag-*Tuba1α* were constructed and co-transfected into mouse fetal fibroblast cells. Fluorescent signals were not observed in the negative control; however, strong signals were visualized in the positive control. Cells co-transfected with pHA-*Tf2b* and pFlag-*Tuba1α* plasmids displayed fluorescent signals, which suggests that TFIIB and α-tubulin directly interact (As shown in Figure S1 in File S1).

### RNAi of *Tf2b* decreased the mRNA level but did not affect nuclear maturation

**Figure 5:**
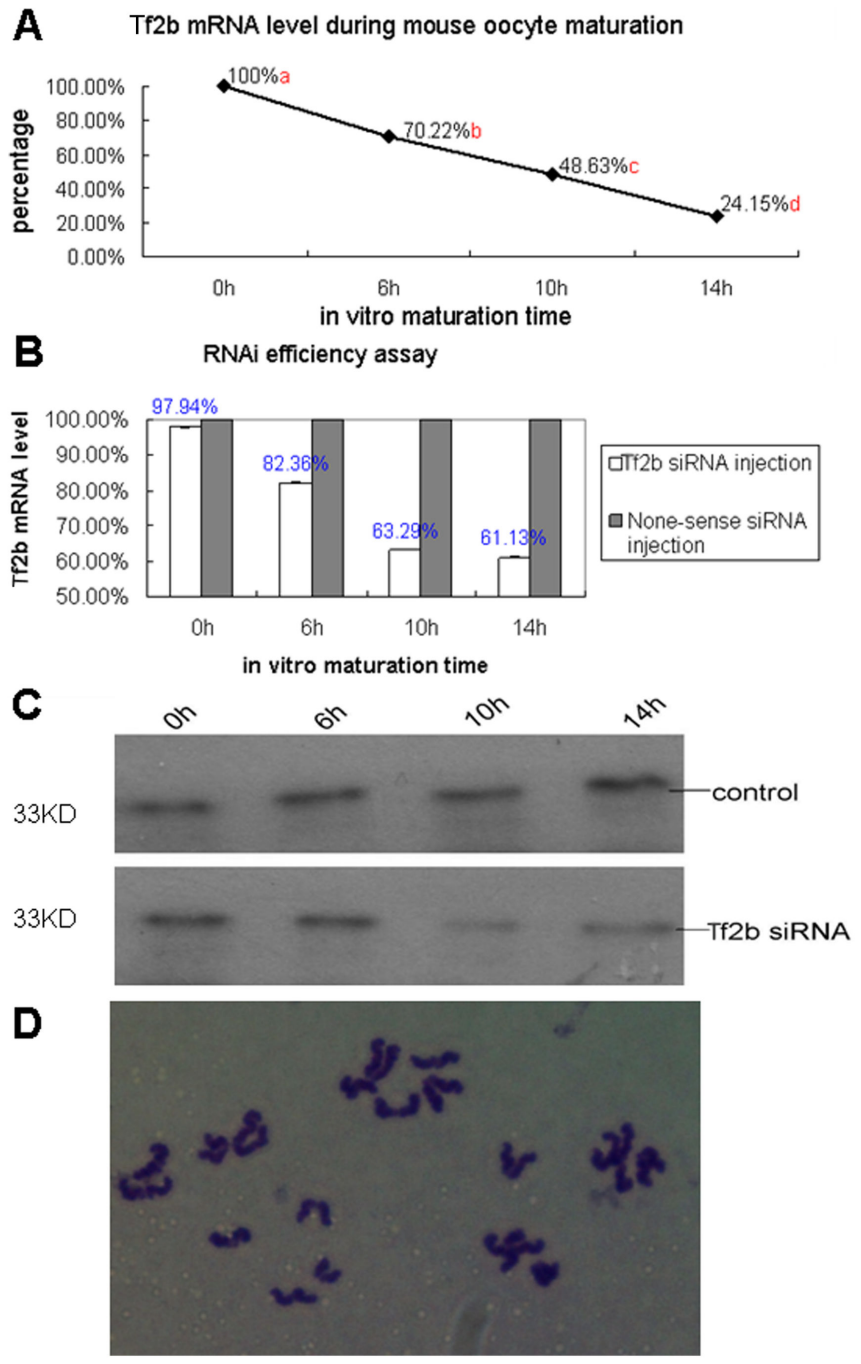
RNAi of *Tf2b* resulted in a decrease of *Tf2b* mRNA, induced irregular spindle formations and chromosome alignments. **A**) *Tf2b* mRNA levels in the maturing mouse oocytes. Oocytes matured to 0, 6, 10, and 14h corresponding to GV, MI, AI/TI, and MII stages, respectively. Different superscripts denote differences (p < 0.01). **B**) *Tf2b* mRNA level after RNAi of oocytes at different stages. **C**) TFIIB expression level during mouse oocyte maturation after RNAi. The molecular mass of TFIIB is 33 kDa. **D**) The MII oocytes derived from GV-injected oocytes had normal chromosome composition (n=20).

After RNAi treatment, oocytes were matured at 0, 6, 10, and 14h to correspond to GV, MI, AI/TI, and MII stages, respectively. Real-time RT-PCR showed that the *Tf2b* mRNA level significantly decreased from 100% at the GV stage to 70.2% at MI, 48.6% at AI/TI, and 24.1% at MII (Figure 5A, B). Western blotting analysis indicated that the *Tf2b* expression significantly decreased upon interference for 6h at MI (Figure 5C). Nuclear maturation of the *Tf2b*-depleted oocytes was not affected. The PB1 extrusion rates were 80.2% (146/182), 82.3% (153/186) and 86.5% (173/200) after *Tf2b* siRNA injection in the non-sense siRNA injection, non-injection and control groups, respectively. The MII oocytes derived from GV-injected oocytes yielded 91.3% (42 /46) with normal chromosomal compositions (Figure 5D). 

### RNAi of *Tf2b* in GV oocytes affected subsequent embryo development and induced developmental arrest

**Figure 6:**
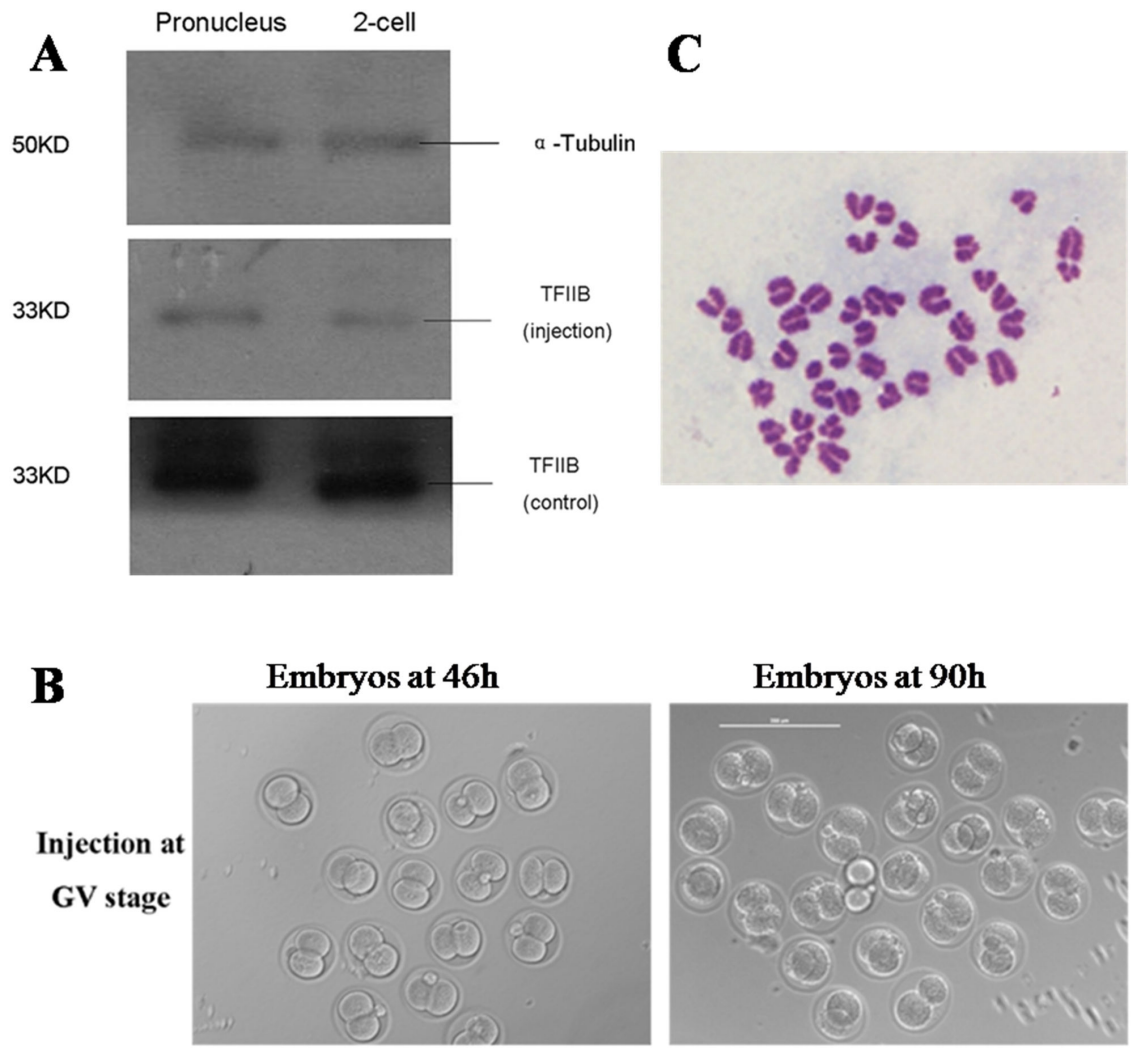
RNAi of *Tf2b* in GV oocytes affected subsequent embryo development. **A**) Expression of TFIIB in mouse early embryos after siRNA injection at the GV stage. The molecular mass of TFIIB is 33 kDa, and the molecular mass of α-tubulin is 50 kDa. **B**) *Tf2b* siRNA injected in the GV stage oocytes arrested mouse embryonic development at the 2-cell stage. Bar=200μm. **C**) Chromosomal composition analysis of the 2-cell embryos derived from GV-injected oocytes (2n=40).

After injection of *Tf2b* siRNAs or non-sense siRNAs into GV oocytes, the oocytes were incubated for 14h. Oocytes with PB1 were then parthenogenetically activated and the embryos were cultured. Real-time RT-PCR showed that siRNA-injected embryos displayed a significantly lower expression than the non-sense injected embryos (Table 3). Western blotting analysis showed that siRNA-injection resulted in significantly decreased TFIIB expression when compared to the non-sense injection group (Figure 6A). 

**Table 3:** The level of *Tf2b* mRNA after *Tf2b* siRNA injection of GV oocytes.

Embryo stages		Ct value of *Tf2b* mRNA	Ct value of *Gapdh* mRNA	2^-ΔΔCt^
MII	*Tf2b* siRNA injection	25.30±0.23	23.26±0.21	100%
	None-sense siRNA injection	26.47±0.23	24.45±0.72	99.8%
Pseudo-Zygote	*Tf2b* siRNA injection	25.02±0.15	24.46±0.04	46.98%
	None-sense siRNA injection	24.48±0.10	25.01±0.17	100%
2-cell	*Tf2b* siRNA injection	25.27±0.17	24.57±0.27	35.60%
	None-sense siRNA injection	23.99±0.33	24.78±0.27	100%
4 days arrested 2-cells	*Tf2b* siRNA injection	25.90±0.21	25.81±0.11	31.64%
	None-sense siRNA injection	24.46±0.24	24.46±0.24	100%

There were no differences in embryo cleavage rates between the *Tf2b* siRNA (67.8%), non-sense siRNA injected (68.0%) and the control (72.9%) groups. The ratio of embryos that developed to the 8-cell and to the blastocyst stages in the *Tf2b* RNAi injection groups were significantly lower than those in the non-sense siRNA injection and control groups (Table 4). More than 85% of the oocytes exhibited a block after *Tf2b* RNAi injection and did not develop beyond the 2-cell stage, although the arrested embryos maintained normal morphology for at least 96h without any apparent degeneration (Figure 6B). The arrested 2-cell embryos in the non-sense RNAi and control groups started to degenerate after 24h. In the 2-cell embryos derived from GV-injected oocytes, 47 cells from 51 embryos were analyzed for their chromosomal composition. Thirty-five cells (74.5%) displayed a normal chromosomal composition (2n=40) (Figure 6C).

**Table 4:** Effects of *Tf2b* RNAi treatment of GV oocytes on subsequent parthenogenic development.

Classes	No. GV oocytes	Maturation rates (%)	No. of 2-cell embryos (%)*	No. of 8-cell embryos (%) **	No. of blastocysts (%) ***
Control	240	203 (84.6)^a^	148 (72.9)^a^	114 (76.9)^a^	103 (69.6)^a^
*Tf2b* siRNA injection	211	171 (81.1)^a^	116 (67.9)^b^	23 (19.7)^b^	13 (11.1)^c^
None-sense siRNA injection	218	178 (81.6)^a^	121 (68.0)^b^	88 (72.7)^a^	71 (65.3)^a^

* Different superscripts within columns denote differences (p < 0.05);

** Different superscripts within columns denote differences (p < 0.01);

*** In the same column, values with different superscripts differ significantly. a, b and b, c: P < 0.01; a, c: P<0.05.

### RNAi of *Tf2b* in MII stage oocytes did not affect embryo development

**Figure 7:**
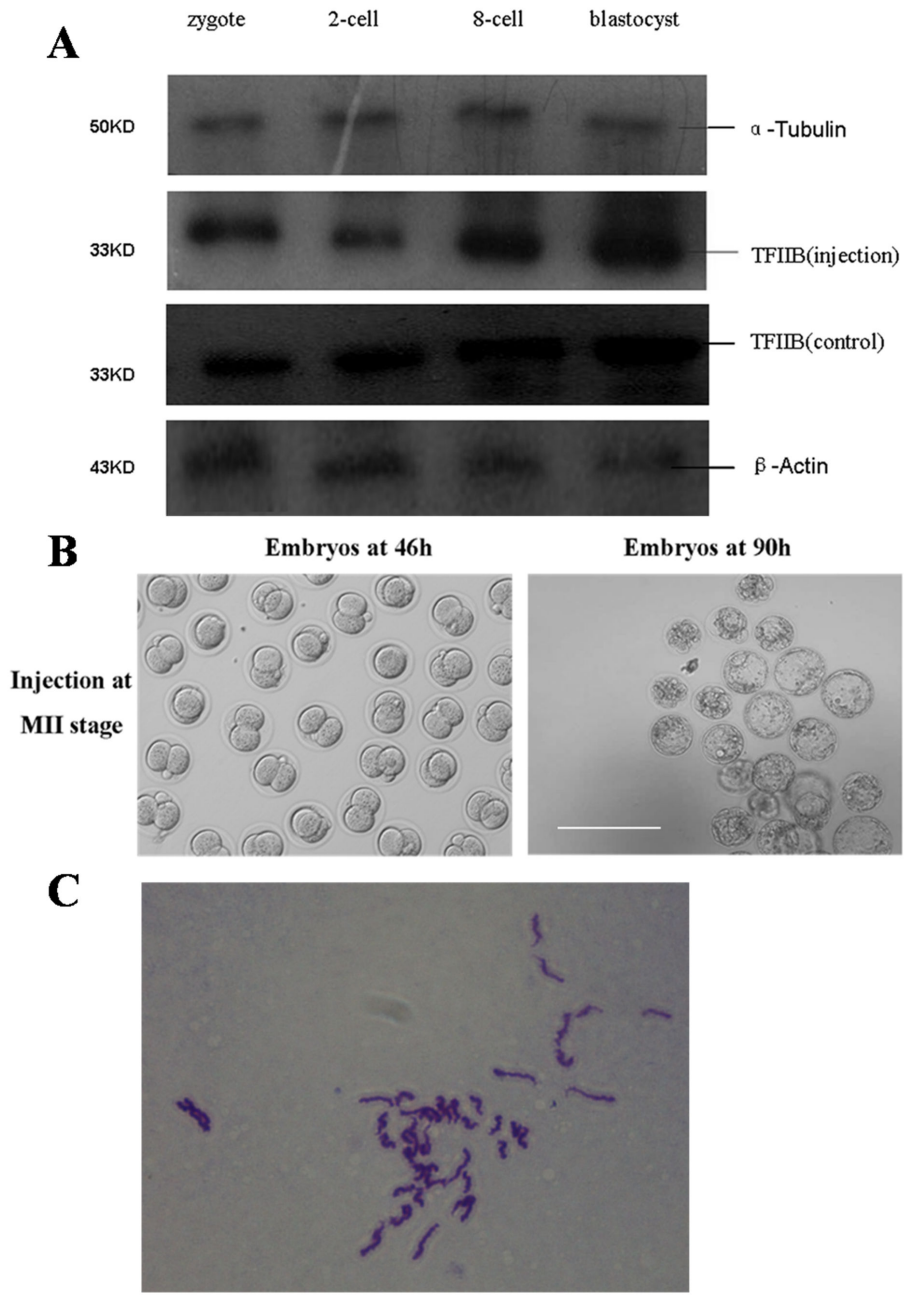
RNAi of *Tf2b* in MII stage oocytes did not affect embryo development. **A**)TFIIB expression in mouse embryos after RNAi treatment. The molecular mass of TFIIB, α-tubulin and β-actin is 33 kDa, 50 kDa and 43 kDa, respectively.B) *Tf2b* siRNA injected in MII oocytes didn’t affect mouse embryonic development. Bar=200μm. **C**) Chromosomal composition analysis of the blastocysts derived from MII-injected oocytes (2n=40).

After *Tf2b* siRNA injection followed by activation and incubation of the MII oocytes, the mRNA levels of *Tf2b* in MII oocytes, pseudo-zygotes, 2-cell, 8-cell, and blastocyst embryos yielded 99.3%, 80.1%, 62.0%, 79.5% and 82.4%, respectively, when compared to the non-sense RNAi groups (Table 5). The lowest mRNA level was detected in 2-cell embryos. Similar observations were observed by Western blotting analysis (Figure 7A). There were no differences in cleavage rates, 8-cell and blastocyst rates among the *Tf2b* RNAi, non-sense RNAi and control groups (Table 6). *Tf2b* siRNA injection of MII oocytes did not affect embryonic development (Figure 7B). In blastocysts derived from MII-injected oocytes, 75 cells from 11 blastocysts were analyzed for their chromosomal composition. Sixty-four of the cells (85.3%) displayed a normal chromosomal composition (2n=40) (Figure 7C). 

**Table 5:** Interference efficiency of mouse *Tf2b* siRNA injected into oocytes at the MII stage.

Groups		Ct value of *Tf2b* mRNA	Ct value of *Gapdh* mRNA	2^-ΔΔCt^
MII	*Tf2b* siRNA injection	23.99±0.57	27.37±0.29	99.31%
	None-sense siRNA injection	25.10±0.50	24.49±0.27	100%
zygote	*Tf2b* siRNA injection	24.03±0.18	24.45±0.29	80.11%
	None-sense siRNA injection	24.73±0.21	25.47±0.20	100%
2-cell	*Tf2b* siRNA injection	22.89±0.44	24.38±0.63	61.99%
	None-sense siRNA injection	22.44±0.52	24.62±0.20	100%
8-cell	*Tf2b* siRNA injection	26.10±0.34	25.84±0.98	79.55%
	None-sense siRNA injection	25.01±0.19	25.08±0.62	100%
Blastocyst	*Tf2b* siRNA injection	26.97±0.15	27.22±0.61	82.36%
	None-sense siRNA injection	25.30±0.18	25.83±0.22	100%

**Table 6:** Effects of *Tf2b* RNAi treatment of MII oocytes on subsequent parthenogenic development.

Groups	No. oocytes	No. 2-cell embryos (%)	No. 8-cell embryos (%)	No. blastocysts (%)
Control	200	167 (83.5)	134 (80.3)	123 (75.3)
*Tf2b* siRNA injection	151	120 (79.5)	98 (81.7)	78 (64.7)
None-sense siRNA injection	148	113 (76.3)	91 (80.3)	78 (68.8)

## Discussion

Following a coordinated series of nuclear and cytoplasmic events, oocytes still maintained their ability to become fertilized with the potential to fully develop [19,21]. The occurrence of GVBD and PB1 extrusion marks the point of nuclear maturity. Spindle microtubules assembled from the opposite poles of the spindle are primarily responsible for chromosomal alignment, segregation and PB extrusion [16,17,22]. Cytoplasmic maturation is a highly complex process that includes meiotic apparatus assembly and peripheral migration, cortical granule redistribution, changes in transcription, protein translation and post-translational modifications [18,23]. Polar body extrusion does not in itself indicate that the cytoplasm has the ability to support embryo development to term. 

Transcription factors gradually disassemble from the chromatin/chromosome as the oocyte progresses from GV to MII. Sun et al. [13](2007) reported in the mouse that within the non-surrounding nucleolus (NSN) and nuclei, transcription is activated by the presence of eight general transcription factors (GTFs) TBP, TRF3, TAF1, TAF4, TFIIA, TFIIB, PolII, and BRF1. Transcription is silenced in SN nuclei. In studies where we used mouse and bovine oocytes as models, we observed that the chromatin factors TAFIIP250, HDAC1, HDAC2, HP1α, HP1β and Topo IIα were associated with the DNA template during chromosomal condensation. BAF155, Brg-1, TFIID (TBP) and Topo Iiβ, were dispersed throughout the nucleoplasm (authors' unpublished data). 

TFIIB is a general transcription factor and it is a target for transcriptional activator proteins that stimulate the preinitiation complex assembly and serves as a bridge between promoter-bound TFIID and RNA polymerase II [7,24]. The localization and/or attachment matrices of TFIIB in cytoplasts have not yet been clearly elucidated. This study focused on the interrelationship and interaction between TFIIB and α-tubulin. TFIIB during oocyte maturation is distributed entirely in the nuclear area of the GV after gradually disassociating from the chromatin. The dissociated TFIIB attaches to the spindle apparatus from GVBD to MII as shown in Figure 2. Colcemid treatment disrupts the microtubule organization, and the TFIIB signals then remain with the altered MTs. Co-transfection of BiFC plasmids pHA-*Tf2b* and pFlag-*Tuba1α* in somatic cells confirmed the direct interaction of TFIIB and α-tubulin (as shown in Figure S1 in File S1). The injection of TFIIB antibodies and siRNA in oocytes verified that the depletion of TFIIB/*Tf2b* causes abnormal spindle formation and irregular chromosomal alignment. These observations suggest that the assembly and disassembly of TFIIB are associated with, and driven by, the microtubules. TFIIB maintains contact with and forms a co-localization distribution pattern with α-tubulin.

Following fertilization and parthenogenetical activation, two phases of transcriptional activation lead to a transition from maternal to the zygotic control of gene expression. The first phase occurs during pronuclear formation of the zygote. The second phase begins at the late 2-cell stage [25]. The present study shows that RNAi significantly reduces *Tf2b* expression, but it does not inhibit PB1 extrusion. Embryo development following treatment of the oocytes was dramatically affected. More than 85% of the embryos did not develop beyond the 2-cell stage. The time point at which embryo development arrested coincided with the occurrence of mouse MZT, which begins at the early 2-cell stage. As discussed above, RNAi of GV oocytes did not affect oocyte nuclear maturation. However, the decrease in the rate of embryo development and the higher incidence of developmental arrest indicates that RNAi of *Tf2b* in GV oocytes affects cytoplasmic maturation and thereby induces developmental arrest. Analysis of the matured oocytes and the resultant embryos suggests that the interference with the GV stage and MII stage oocytes had little effect on the chromosomal composition of oocytes/embryos. The matured oocytes displayed a haploid composition and the blastocysts derived from the MII-injected oocytes were diploid. The fact that RNAi of MII oocytes did not influence embryo development suggests that MII oocytes probably accumulate enough required materials for subsequent embryo development before the interfering affects of *Tf2b*. This observation further supports in principle that the depletion of *Tf2b* in GV oocytes affects oocyte cytoplasmic maturation, especially the transcriptional activation activities that lead to the failure of embryonic MZT. 

It is intriguing that the arrested 2-cell embryos maintained normal morphology and appeared to be viable for at least 96 h with no apparent signs of degeneration, whereas the 2-cell arrested embryos in the control and nonsense-injected groups started to degenerate at around 24h. For now, we have no reasonable explanation for why an embryo in an arrested state will maintain an appearance of being viable for a prolonged period of time. In mouse zygotes, transcription activity in pronuclear oocytes represents 40% of the transcriptional level observed at the 2-cell stage [26-28]. Zernicka-Goetz et al (2006) reported in mouse embryos that the transcription intermediary factor 1α (TIF1α) is involved in modulating expression of a group of genes during the first wave of genome activation [29]. What may be occurring in the prolonged 2-cell arrested embryos is an alteration in the expression of TIF1α that slows the degenerative process in arrested embryos.

Based upon the data generated in this study, we conclude that during oocyte meiosis in the mouse, TFIIB co-localizes with α-tubulin, and that the assembly and disassembly of TFIIB from the chromatin during transcription cession and initiation appears to be driven by α-tubulin. The depletion of *Tf2b* not only decreases *Tf2b* expression, but it also affects the TFIIB-associated microtubules, which then results in irregular spindles. The ablation of TFIIB has no effect on oocyte nuclear maturation, but did affect cytoplasmic maturation. RNAi of *Tf2b* resulted in the majority of embryos arresting at the 2-cell stage, with the arrested embryos having a prolonged period of survival. The results of this study imply that the interruption of TFIIB in GV oocytes affect genes or factors associated with MZT, which then leads to the arrest of embryo development. 

## Supporting Information

File S1 **BiFC analysis of mitotic cells revealed the direct interaction between TFIIB and α-tubulin.** Figure S1 in File S1, Micrographs of BiFC analysis.
(DOC)
