# Supplementary material for: TFIIB Co-Localizes and Interacts with α-Tubulin during Oocyte Meiosis in the Mouse and Depletion of TFIIB Causes Arrest of Subsequent Embryo Development
Source: PLoS One. 2013 Nov 14;8(11):e80039. doi: 10.1371/journal.pone.0080039 (PMC3828216; doi:10.1371/journal.pone.0080039)
Supplement: File S1 — BiFC analysis of mitotic cells revealed the direct interaction between TFIIB and α-tubulin. Figure S1 in File S1, Micrographs of BiFC analysis. (DOC) [file pone.0080039.s001.doc]

Supplemental materials

**Construction of plasmids for the BiFC assay**

The plasmids for the BiFC were constructed as described ^[1]^. Briefly, total RNA was extracted from 30 milligrams liver tissue of BDF1 mouse using Aqua-SPIN RNA Isolation Micro Kit (Watson). The first strand cDNA was generated using [PrimeScript One Step RT-PCR Kit Ver.2](http://www.takara.com.cn/?action=Page&Plat=pdetail&newsid=294&subclass=1)(Takara) with poly (dT) primers during 15 min at 37 ^o^C, followed by 5 sec at 85^o^C. The following two primers were used to clone the full length of *tf2b* and α-*tubulin* cDNA by PCR respectively, which was designed by software Primer Premier5.0 according to the sequences in Genbank.

tf2b (NM_145546) forward 5’- TCGGAATTCTTGGCGGAGCCGCGAAGATG-3’, tf2b (NM_145546) reverse 5’- CTCGGTACCTAGCTGGGGTAATTTGTCCAC-3’; α-tubulin (NM_011653) forward 5’- ACTAAGCTTCGCGAAGCAGCAACCATGCGTGAGT-3’, α-tubulin (NM_011653) reverse 5’- GCATCTAGAGTATTCCTCTCCTTCTTCCTCCCCC-3’.

The recognized sequence of restriction enzyme E*coR* I and K*pn* I was added to the 5’end of tf2b sense and anti-sense primer respectively. The recognized sequence of H*ind* III and X*ba* I was designed to the 5’end of α-*tubulin* sense and anti-sense primer respectively. After subcloning into the pUC19 vector (Takara, Dalian, China), the E*coR* I–K*pn* I fragment containing the *tf2b* encoding sequence was ligated into the corresponding sites of plasmid pHA-VC155. The H*ind* III–X*ba* I fragment containing the α-tubulin encoding sequence was ligated into the corresponding sites of plasmid pFlag-VN173. The resulting plasmids were identified as pHA-tf2b and pFlag-tuba1α, respectively. All constructs used in this investigation were verified by DNA sequencing. E. coli DH5α served as host for general plasmid construction and maintenance. Positive control plasmids were pHA-bFos-VC155 and pFlag-bJunVN173; negative control plasmids were pHA-bFos delta-VC155 and pFlag-bJunVN173.

**Cell culture and transfection**

Mouse embryo fibroblast cells（MEFs）were prepared using standard procedures^[2]^ and cultured in DME medium supplemented with 10% FBS, 60µg/ml penicillin and 50µg/ml streptomycin. Plasmids pHA-bFos-VC155 and pFlag-bJunVN173, pHA-tf2b and pFlag-tuba1α, pHA-bFosdelta-VC155 and pFlag-bJunVN173 were co-transfected respectively to MEFs using Lipofectamine LTX and PLUS Reagents (Invitrogen) according to the manufacturers standard procedures. Fluorescence was examined 24h after transfection.

**TFIIB and α-tubulin has interactions in MEFs**

The result showed that none fluorescent signals were observed in negative control but strong signals were found in the positive control. Co-transfection of pHA-tf2b and pFlag-tuba1α resulted cells with fluorescent signals which suggested TFIIB and α-tubulin interacted directly (Fig. S1).


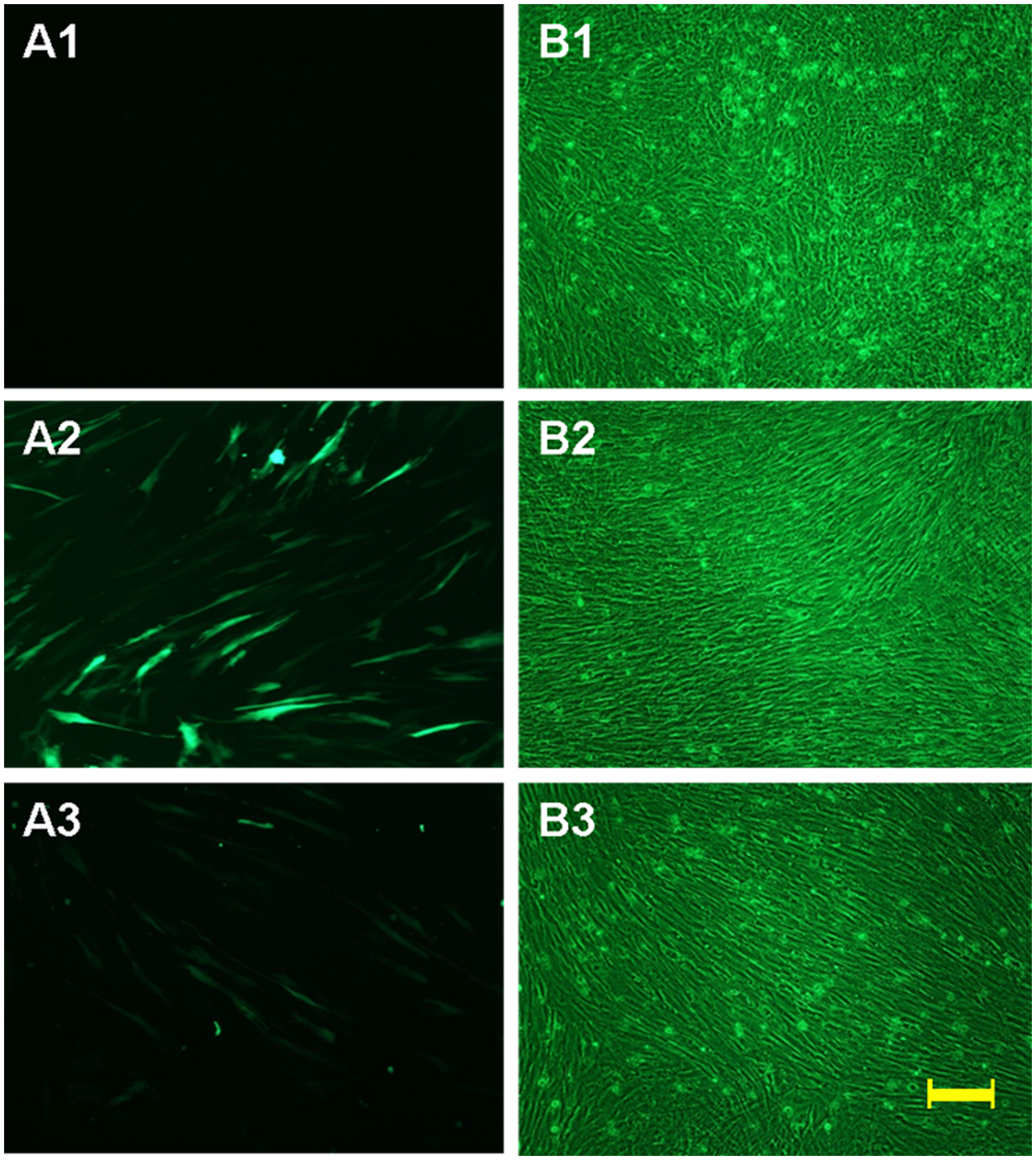


**S1. Micrographs of BiFC analysis.** A1: negative control, co-transfected with plasmids pHA-bFosdelta-VC155 and pFlag-bJunVN173; A2: Fluorescent image of MEF cells co-transfected with plasmids pHA-bFos-VC155 and pFlag-bJunVN173; A3: Fluorescent microscope images of MEF cells co-transfected with plasmids pHA-tf2b and pFlag-tuba1α; B1-B3: White light images of MEF cells in the same region of A1-A3, respectively. Bar=50μm.

**References**

[1] Abedi M, Caponigro G, Shen J, [Hansen S](http://www.ncbi.nlm.nih.gov/pubmed?term=Hansen S%5BAuthor%5D&cauthor=true&cauthor_uid=11580863), [Sandrock T](http://www.ncbi.nlm.nih.gov/pubmed?term=Sandrock T%5BAuthor%5D&cauthor=true&cauthor_uid=11580863), *et al*. (2001)Transcriptional transactiveation by selected short random peptides attached to lexA-GFP fusion proteins. BMC Mol Biol 2(10): 1-10.

[2] Bryja V, Bonilla S, Arenas E. (2006) Derivation of mouse embryonic stem cells. [Nat Protoc](http://www.medsci.cn/sci/hotlight.asp?id=cbde7617). 1(4): 2082-2087.
